# Tuberculosis Endometritis Presenting as A Leiomyoma

**DOI:** 10.22074/ijfs.2015.4187

**Published:** 2015-02-07

**Authors:** Mahboobeh Shirazi, Fatemeh Shahbazi, Leila Pirzadeh, Seyed Rahim Mohammadi, Parisa Ghaffari, Tahereh Eftekhar

**Affiliations:** 1 Maternal, Fetal and Neonatal Research Center, Vali-Asr Hospital, Tehran University of Medical Sciences, Tehran, Iran; 2 Department of Biology, Payame Noor University, Iran; 3 Obstetrics Gynecology, Gynecology Ward, Emam Hospital, Keshavarz blvd, Tehran, Iran

**Keywords:** Genital Tuberculosis, Leiomyoma, Iran

## Abstract

Genitourinary tuberculosis is a common extrapulmonary manifestation of tuberculosis. Taking into consideration that genitourinary tuberculosis may be associated with a diversity of presentations, its diagnoses may be difficult. A young woman with an initial presumptive diagnosis of a uterine leiomyoma presented with abdominal pain and a pelvic mass that after further investigations, she was diagnosed with genital tuberculosis.

## Introduction

Female genital tuberculosis (TB), following lymphatic tuberculosis, is the second most common extra pulmonary manifestation of tuberculosis (1).

Signs and symptoms of pelvic TB may be diverse and nonspecific, including chronic lower abdominal/ pelvic pain, abdominal/pelvic masses, anorexia, weight loss, fever, abnormal uterine bleeding and infertility. Moreover, an elevated serum CA125 level, leukocytosis, and anemia may also be detected in patients having genital tuberculosis (2, 3). Female genital TB occurs in relatively young females in the reproductive age group (4). Hatami’s study showed that the most commonly affected age group is in range of 26- 30 (5).

We report a case of a 25-year-old woman with genital tuberculosis mimicking a uterine leiomyoma.

## Case Report

A 25-year-old Iranian G2P1Ab1L1 woman was admitted with a 4 month history of weight loss, weakness, anorexia and dull abdominal pain in the hypogastria and left lower quadrant occasionally radiating to the lumbar region. She had a history of one abortion and two operations, cesarean section and appendectomy.

There was no history of infertility, abnormal uterine bleeding, dysmenorrhea, dyspareunia, fever, cough, dyspnea, nausea and vomiting, urinary or gastrointestinal complications. Her medical and family history was unremarkable. The patient was not infertile and her contraception was withdrawal. Furthermore, the patient had also received her childhood bacille Calmette- Guerin (BCG) vaccination. The patient was pale and in her physical examination, we found only a mild to moderate abdominal tenderness in the left lower quadrant and hypogastric region. On further examination, a normal size mid-position uterus with a 6-7 cm palpable mass posterior to the uterus was detected, in which the left ovary was impossible to be detect. The right ovary was palpable and cervical motion tenderness was negative.

Laboratory tests showed only a mild anemia (Hb=10.5 mg/dL) and the other hematologic, biochemical, viral and tumor markers [including cancer antigen (CA)-125, alpha-feto-protein, carbohydrate antigen 19-9, carcinoembryonic antigen (CEA), and lactate dehydrogenase (LDH)] were normal. Furthermore, radiologic investigations of the chest and lumbar spine were also normal. HIV testing was negative in this patient. Furthermore, radiologic investigations of the chest and lumbar spine were also normal.

**Fig 1:**
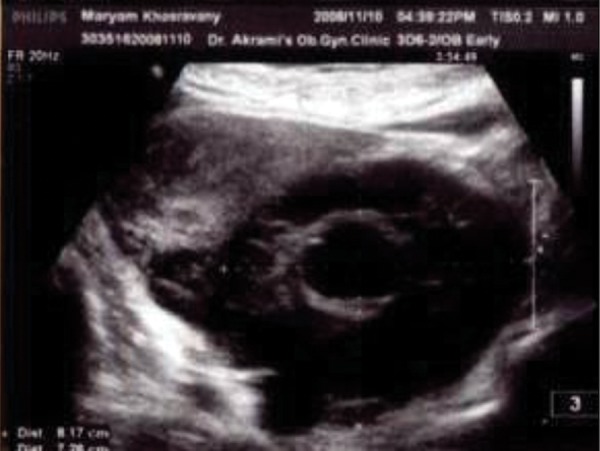
Represents the uterus and mass on the posterior with a hypoechoic pattern in the mass.

Abdominal and vaginal ultrasonography showed the right ovary and uterus to have a normal size and shape. However, there was a heterogenic solid mass (110 cm ×64 cm ×8.7 cm) lying posteriorly between the uterus and left ovary (Fig 1). The ultrasonographic image with standard view was impossible due to frozen pelvic. The vascular pattern of the mass was dominant, only having a simple cyst 2 cm ×3 cm in dimension. The hypoechoic pattern in the mass was suspicious for degenerated leiomyoma. There was no free fluid found in the abdominopelvic cavity. Because of abdominal pain, an exploratory laparotomy was performed.

During the procedure, no seeding or ascites were found. However, there were severe adhesions among the bowel loops, omentum, dilated fallopian tubes and uterus. A necrotic mass (7 cm×6 cm) in the posterior wall of the uterus was seen. The left dilated tube and ovary were adherent to the posterior wall of the uterus and multiple biopsies were sent for frozen section.

**Fig 2:**
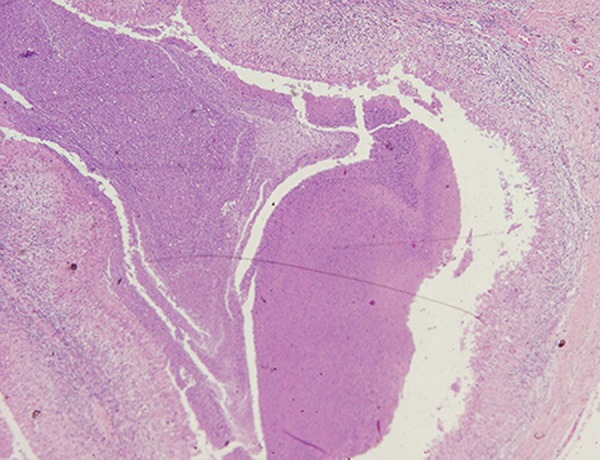
Represents the granulomatous reaction and central necrosis (×40). Epithelioid cells and mixtures of other cells, including epithelioid macrophages, giant cells (Langhans type giant cells in which the nuclei are lined up around the periphery of the cell), lymphocytes, plasma cells, and fibroblasts, surround a central area of necrosis that appears irregular, amorphous, and pink. There may be some neutrophils.

Caseous necrosis, devoid of malignant cells, was seen in the biopsy of mass using hematoxylin and eosin staining (Fig 2). Peritoneal fluid and sample were stained, specially using the Ziehl-Neelsen staining technique. Peritoneal washings and a number of biopsies were sent in for culture.

*Mycobacterium tuberculosis* was visualized after 5 weeks using Lowenstein-Jensen medium that confirmed the diagnosis of genital tuberculosis.

The therapy was started empirically according to the histopathological results.

The patient underwent a 9 month course of quadruple anti-TB therapy including isoniazid [isonicotinic acid hydrazide (INH)], rifampicin (RFP), ethambutol and pyrazinamide. The patient responded well to the treatment and during a 2 year follow-up, no relapse was detected.

## Discussion

TB causes about 3 million deaths worldwide each year (6). Genital TB, following lymphatic tuberculosis, is the second-most common extra pulmonary manifestation of tuberculosis and is more common among females (1, 7).

The clinical findings of genital TB are nonspecific, some of the constitutional symptoms are weight loss, anorexia, sweat and fever.

Most of the patients may be asymptomatic; however, three major complaints have been reported which include infertility (65-70%), abdominal/ pelvic pain (50-55%) and menstrual abnormalities (20-25%) (2). Our patient did not experience infertility.

Serum CA125 may be elevated in genital TB (8-10). Therefore, it might mimic ovarian cancer, endometriosis, Meigs syndrome, ovarian hyperstimulation, etc. Other serum markers have limited value and other tests, such as ultrasonography, and computed tomography may suggest ovarian malignancy, tuboovarian mass (TOA), ectopic pregnancies and leiomyomas (3).

The diagnosis of genital TB can be done with fine needle aspiration by detecting caseous granulomas or acid fast bacilli in the smears (11). In 50-60% of genital TB, the endometrium is involved (3, 7). Similar to the Xi’s study (12), examination of ascetic fluid was negative using the Ziehl-Neelsen staining technique. Biopsies from the lesions via laparotomy or laparoscopy can also help the diagnosis of genital TB. A definitive diagnosis is based on a Ziehl-Neelsen staining for acid fast bacilli, a positive culture, or polymerase chain reaction (PCR) of the *Mycobacterium-tuberculosis* gene which has a high sensitivity and specificity (82- 86 and 95%, respectively) and its results are more rapid when compared to the culturing of the bacterium (2 days instead of weeks) (13). This particular case was interesting in the sense that the patient had only suffered from weight loss, anorexia and abdominal pelvic pain. All lab tests, apart from a mild anemia, were normal and the ultrasonography only suggested a leiomyoma or ovarian tumor.

In conclusion, the diagnosis of genital TB should be considered in all women with pelvic masses and constitutional symptoms and signs, especially in endemic areas like Iran. Consequently, medical therapy is recommended for advanced genital TB. If the patient does not respond to medical therapy, a total abdominal hysterectomy with bilateral saplingo-oophorectomy is recommended.
